# A Validated LC–MS/MS Method for Quantifying Phenolic Acids, Lignans, and Enterolignans from Human Fecal Samples

**DOI:** 10.1021/acsnutrsci.6c00063

**Published:** 2026-06-11

**Authors:** Christopher A. Dicksion, Darrian N. Chao, Jonathan D. Rickmeyer, Elizabeth N. Bess

**Affiliations:** † Department of Chemistry, 8788 University of California, Irvine, California 92617, United States; ‡ School of Pharmacy and Pharmaceutical Sciences, 8788 University of California, Irvine, California 92617, United States; § Department of Molecular Biology and Biochemistry, 8788 University of California, Irvine, California 92617, United States

**Keywords:** LC−MS/MS, human gut microbiome, phenolic acids, lignans, enterolignans, fecal samples

## Abstract

The human gut is home to numerous small molecules that impact health. Three prominent classes of molecules in this environment are phenolic acids, lignans, and enterolignans, which have been linked to anti-inflammatory and antioxidant effects as well as protection from cancer, cardiovascular disease, and neurodegeneration. The abundance of these molecules in the intestine as well as their biological significance motivated the development and validation of the LC–MS/MS method reported herein, which provides a simple, robust, and high-throughput approach to simultaneously quantify a 22-membered panel of phenolic acids, lignans, and enterolignans in human fecal samples. Facile sample preparation and a short analytical time (5 min per sample, compared to similar methods that range from 7.8–28 min) allow for high throughput. A 16-fold increase in sensitivity allows for quantitation of lignans that are often not detected via existing methods.

## Graphical abstract

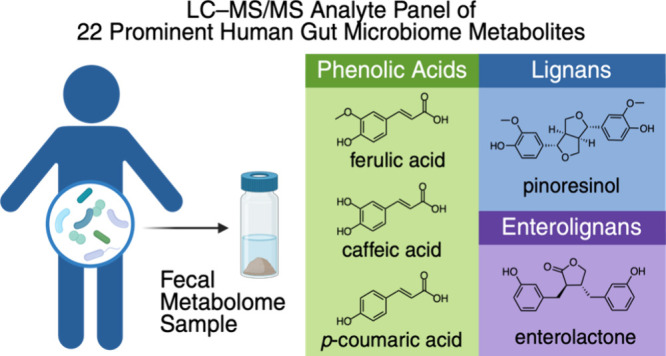

## Introduction

Metabolites produced by bacteria in the human gut are emerging as critical modulators of human health.^1,2^ As such, the need for analytical methods to identify and quantify metabolites produced by gut bacteria is critical for studies that seek to link metabolomic profiles to the development, progression, and treatment of disease.^3–5^ Equally important is the development of methods for the quantitation of health-promoting gut microbial metabolites, such as the diverse class of polyphenols, including phenolic acids, lignans, and enterolignans.^6^ These compound classes contain molecules that exert antioxidant,^7^ anti-inflammatory,^8^ antihypertensive,^9^ and anticancer effects,^10^ highlighting their potential as therapeutic agents against a variety of diseases. Phenolic acids and lignans are biosynthesized in plants from the same molecular precursors,^11^ and enterolignans are synthesized from lignans by human gut microbiota.^12^ To facilitate studies that seek to identify new links between gut bacterial metabolism and human health, we have established a validated LC–MS/MS method for the quantification of a 22-membered panel of molecules in human fecal samples that are derived from plant-based diets: 15 phenolic acids, 5 lignans, and 2 enterolignans (Figure 1 and Table S1).

**Figure 1:**
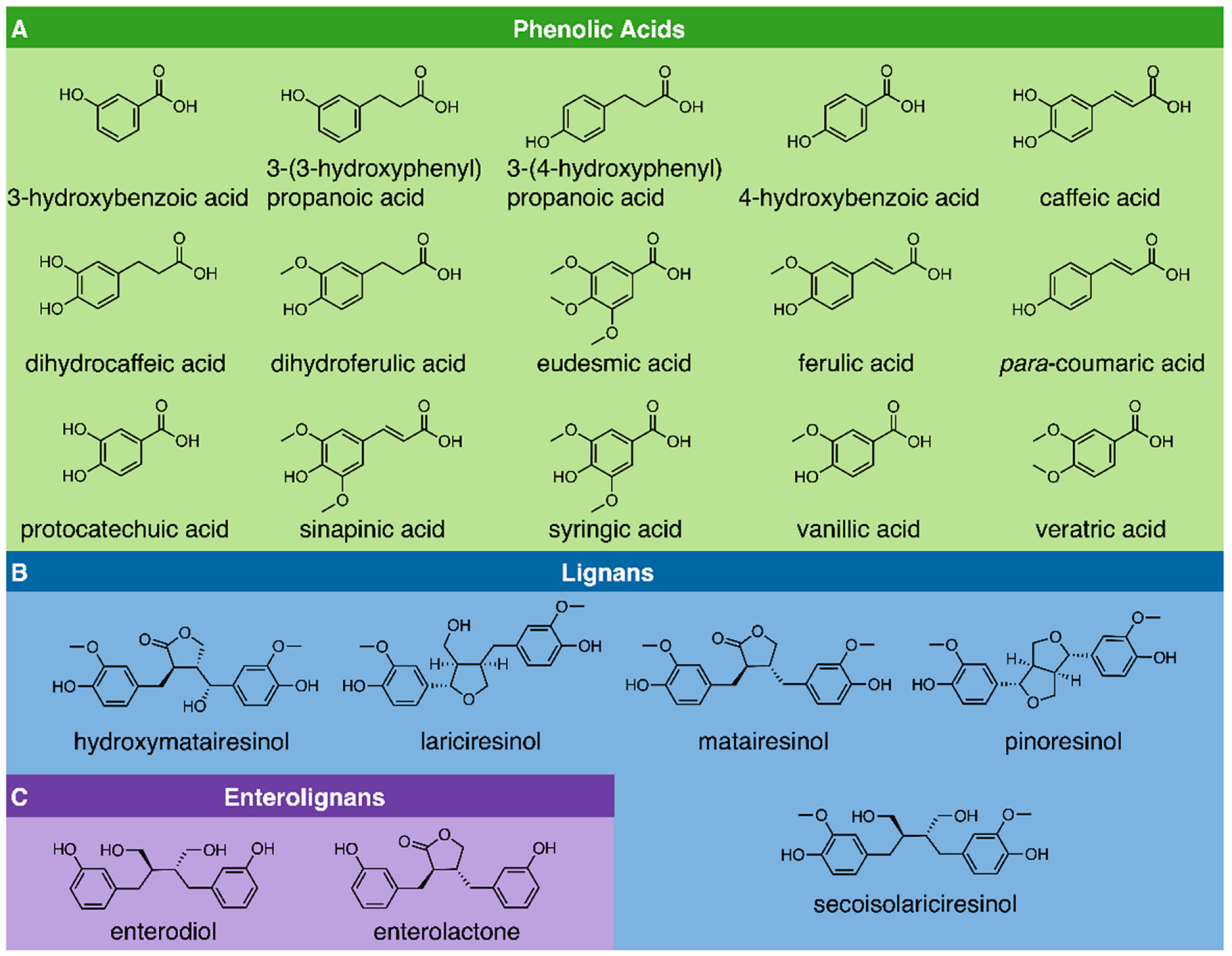
Analyte panel. Collection of (A) 15 phenolic acids, (B) 5 lignans, and (C) 2 enterolignans quantified using the LC–MS/MS method reported herein.

The largest class of molecules measured by our method is phenolic acids (Figure 1A). These plant secondary metabolites are prevalent in fruits, vegetables, and beverages like coffee and tea.^13^ Average dietary intake of phenolic acids among U.S. adults is estimated to be 1005.6 mg per day.^14^ Phenolic acids not only contribute to the flavor, aroma, and nutritional profiles of foods and beverages,^15^ but they also have properties that may fight diseases ranging from cancer to neurodegeneration. For example, Balsamo et al. showed that one of the most diet-abundant phenolic acids, caffeic acid,^16^ significantly limits a Parkinsonian neurodegenerative pathway (i.e., formation of α-synuclein aggregates) in murine intestinal enteroendocrine cells; caffeic acid exerts this effect by suppressing the oxidation of dopamine, the mediator of this pathogenic process.^17^

The next class of molecules measured using the method reported here is lignans (Figure 1B). These dimeric phenylpropanoid metabolites are found in fiber-rich foods, especially seeds and nuts,^18^ with total dietary consumption averaging around 1 mg per day.^19^ Lignans have been shown to exert anticancer effects by inhibiting tumor growth and metastasis in animal models^20^ as well as by inducing apoptosis in breast cancer, colorectal cancer, and prostate cancer cell lines.^21^ Lignans may also improve cardiovascular health. A prospective study of over 200,000 U.S. adults found that increased lignan intake, especially from dietary fiber, was associated with a lower risk of coronary heart disease.^22^

Diet-derived lignans not only exert health-promoting effects in their own right, but they are also metabolized by a consortium of human gut bacteria into the enterolignans, enterodiol and enterolactone (Figure 1C).^23^ Enterolignans, which are quantified via the method reported here, exhibit their own anticancer effects.^10^ When gnotobiotic rat models of breast cancer were colonized with a lignan-metabolizing bacterial consortium and fed a lignan-rich diet, fecal concentrations of enterolactone increased in tandem with tumor-cell apoptosis and overall tumor burden decreased as compared with uncolonized gnotobiotic rats.^24^ Aligned with these findings, studies in the MCF-7 breast cancer cell line show that enterolactone is an aromatase inhibitor that limits proliferation of cancer cells by interrupting the formation of estradiol.^25^ Taken together, these findings suggest that gut microbiome profiles encoding for specific biosynthetic pathways may impact breast cancer risk and improve health outcomes for people living with breast cancer. Indeed, a recent meta-analysis of three observational studies encompassing 3,864 participants concluded that serum enterolactone concentrations around 80 ng/L were correlated with a 28% decrease in mortality from breast cancer.^26^

Due to the health-promoting effects of phenolic acids, lignans, and enterolignans, we selected a panel of 22 analytes that are linked by their biosynthetic origins and sought to quantify each within a single LC–MS/MS method. The targeted LC–MS/MS method presented herein contributes to the science of gut microbiota metabolomics by improving upon existing methods, and it has been validated to conform to established FDA guidelines.^27^ First, whereas other targeted mass spectrometric methods can quantify either phenolic acids, lignans, or enterolignans, the current method can account for analytes from all three classes of compounds in a single injection. The method developed by González-Domínguez et al. quantified molecules from these 3 classes in a single injection, but the sample preparation was optimized for plasma (not fecal samples), the run-time per injection was 14 min, and it was 16- and 26-fold less sensitive for detecting lignans and enterolignans, respectively, than what is achieved by the new method reported here.^28^ Additionally, an important precursor in the enterolignan biochemical pathway, pinoresinol, was not included in this prior report.^28^ Second, our method normalizes analyte concentrations to the mass of dry fecal samples; due to differences in the water content of fecal samples from person to person, normalizing the analyte content to the dry mass of the fecal sample provides a reliable and reproducible way to standardize metabolite abundance comparisons between individuals, across studies, and over time.^29,30^ Lastly, this method contributes enhanced analytical throughput; whereas most existing methods for these analytes have total run-times between 7.8 and 28 min, the run-time per sample for the method reported here is only 5 min.

## Materials and Methods

### Chemicals

Caffeic acid, *para*-coumaric acid, and protocatechuic acid were purchased from Tokyo Chemical Industry, Co., in 98% purity. Ferulic acid (99%), 3-hydroxybenzoic acid (99%), 4-hydroxybenzoic acid (99%), 3-(4-hydroxyphenyl)­propanoic acid (98%), and vanillic acid (97%) were purchased from Sigma-Aldrich. Dihydrocaffeic acid and 3-(3-hydroxyphenyl)­propanoic acid were purchased from Alfa Aesar in 98% purity. Dihydroferulic acid was purchased from Matrix Scientific in 98% purity. Eudesmic acid (99%), sinapinic acid (95%), syringic acid (95%), and veratric acid (98%) were purchased from AK Scientific. (−)-Matairesinol was purchased from Santa Cruz Biotechnology in 95% purity. (−)-Enterodiol was purchased from PhytoLab in 95% purity. (−)-Enterolactone, (−)-hydroxymatairesinol, (+)-lariciresinol, (+)-pinoresinol, and (−)-secoisolariciresinol were purchased from Sigma-Aldrich in 95% purity. The internal standards (IS) ^13^C_3_-caffeic acid, *d*_6_-rac-secoisolariciresinol, and ^13^C_3_-rac-enterolactone were purchased from Toronto Research Chemicals in 95% purity. HPLC grade methyl *tert*-butyl ether (MtBE) as well as LC–MS grade acetonitrile and acetic acid were purchased from Fisher Scientific. Double deionized water (DDI H_2_O) was obtained from an Elga PURELAB flex water purifier at greater than 18.2 MΩ·cm.

### Fecal Samples

Fecal samples were obtained from 10 healthy human volunteers who consented to participate in the study (protocol #20195091), which was approved by the University of California, Irvine Institutional Review Board. Samples were collected using a GutAlive anaerobic microbiome kit (Microviable, Gijón, Spain). Upon receipt, the samples were brought into an anaerobic chamber (Coy Laboratory Products, Grass Lake, MI) with an atmosphere of 20% CO_2_, 2%–5% H_2_, and the balance N_2_. Between 0.5 and 2.0 g of fecal material was mechanically homogenized by mixing with a spatula and then added to 15 mL conical tubes and stored at −80 °C. The frozen samples were lyophilized for 5 days. After lyophilization, 50 mg from each donor were combined to make a fecal mixture (n = 10 donors) for validation experiments. Lyophilized samples were stored at −80 °C until use.

### Calibration and Quality Control Sample Preparation

Internal standards (IS) ^13^C_3_-caffeic acid, ^13^C_3_-rac-enterolactone, and *d*_6_-rac-secoisolariciresinol were individually prepared as 10 mM stocks in acetonitrile, and 10 μL of each were subsequently combined with 970 μL DDI H_2_O to make 100 μM stock solutions. All stocks were stored at −80 °C until use. Working solutions of IS at 100 nM were prepared by adding 100 μL of 100 μM IS mix to 99.9 mL DDI H_2_O containing 5% acetonitrile. Commercially available standards for each analyte in the 22-membered panel were weighed and dissolved in acetonitrile to make 22 individual 10 mM stock solutions. Aliquots of the individual stock solutions were stored at −80 °C until use. These individual stocks were combined to make 1 mL of a 100 μM mixture of each standard in DDI H_2_O and 5% acetonitrile containing the internal standards. Aliquots of the 100 μM stock mixture were stored at −80 °C. Upon use, an aliquot of the 100 μM stock mixture was thawed at room temperature for 5 min before being diluted to 1 μM with DDI H_2_O and 5% acetonitrile containing IS. The calibration samples were prepared as 2-fold serial dilutions from 1000 nM to 0.244 nM in DDI H_2_O and 5% acetonitrile containing IS and were used immediately.

Quality control (QC) samples were prepared at 10 nM, 40 nM, 400 nM, and 800 nM in DDI H_2_O containing IS (100 nM) by adding 600 μL of each QC sample to 2.0 mL glass HPLC vials and capped. The samples were mixed at 1000 rpm for 5 min. After the addition of 1.2 mL MtBE + 0.1% acetic acid, the samples were mixed for 5 min at 1000 rpm. Next, the samples were centrifuged at 3000 rpm for 10 min, and then 900 μL of the MtBE supernatant was transferred to a glass culture tube. The samples were dried under N_2_ for 15 min at 40 °C with a Biotage Turbovap LV (Biotage, Uppsala, Sweden). Dried residues were resuspended in 150 μL DDI H_2_O containing 5% acetonitrile, capped with a butyl stopper, and mixed at 500 rpm for 15 min. The resuspended samples were transferred to low-volume glass HPLC vials and centrifuged for 10 min at 3000 rpm. A 100 μL aliquot of the supernatant was transferred to a 96-well plate and immediately subjected to analysis.

### Method Validation

This method was developed to conform to established FDA guidelines, so each analyte was assessed according to sensitivity, selectivity, matrix effects, stability, accuracy, precision, and recovery.^27^

#### Sensitivity

The sensitivity of the method was established by first recording the area under the curve for each analyte across 12 sequential blank injections, consisting of 5% aqueous acetonitrile. Following this, a 2-fold dilution series from 1000 nM to 0.244 nM of a mixture of the 22 analytes was prepared in DDI H_2_O, and the lower limit of quantitation (LLOQ) was determined to be the lowest concentration in the dilution series that displayed an absolute area under the curve greater than 10 times the average absolute area under the curve in the replicate blank injections.

#### Selectivity

Selectivity was assessed by first injecting a mixture of all the analytes at 1.0 μM in DDI H_2_O containing 5% acetonitrile to establish the retention time of each analyte. Fecal samples (25 mg lyophilized fecal material) were prepared according to the quality control sample procedure (vide supra), injected into the mass spectrometer, and the presence of interfering or overlapping peaks was assessed for each analyte. Cross-signal contributions were assessed by preparing standards individually and as a mixture in 5% aqueous acetonitrile at 1.0 μM.

#### Matrix Effects

Matrix effects were assessed according to the method established by Matuszewski et al.^31^ Samples were prepared in triplicate for each QC concentration, and the method of background subtraction was used to account for the presence of endogenous analytes. Briefly, 25 mg of lyophilized fecal material were added to a 2.0 mL glass HPLC vial. After adding 600 μL DDI H_2_O, the samples were mixed at 1000 rpm for 5 min. To this slurry, 1.2 mL MtBE + 0.1% acetic acid was added, and the samples were mixed again for 5 min at 1000 rpm. After centrifugation at 3000 rpm for 10 min, 900 μL of the MtBE supernatant was transferred to a glass culture tube. The residues were dried under a stream of N_2_ gas at 40 °C for 15 min. The samples were resuspended in 150 μL of DDI H_2_O containing 5% acetonitrile and a mixture of analyte standards at 0 nM (termed Endo), 10 nM, 40 nM, 400 nM, and 800 nM (collectively termed Matrix) and internal standards at 100 nM. The resuspended residues were mixed for 15 min at 500 rpm, then transferred to low-volume glass HPLC vials and centrifuged for 10 min at 3000 rpm. A 100 μL aliquot of the supernatant was transferred to a 96-well plate and used immediately for analysis. To account for endogenous analyte concentrations near or above the upper limit of quantitation (ULOQ), 50-fold dilutions of the resuspended samples were prepared and used immediately. Neat mixtures of the analyte standards at the same concentrations were prepared in DDI H_2_O containing 5% acetonitrile and used for comparison. Results were calculated via background subtraction for each replicate at each concentration as the percent of the analyte response of the spiked samples to the response of neat samples according to eq 1; values less than 100% signify ion suppression and values greater than 100% signify ion enhancement. Results are reported as the mean ± S.E.M.

$$Matrix\;Effect\left(\%\right)=\left(\frac{{Response}_{Matrix}-{Response}_{Endo}}{{Response}_{Neat}}\right)\times 100\%$$ 1

Interferences from leachate of polypropylene microcentrifuge tubes (MCTs) were assessed by adding 600 μL DDI H_2_O ± 0.1% formic acid to two different brands of MCTs and to one brand of glass HPLC vials, followed by liquid–liquid extraction with 1.2 mL MtBE. The samples were mixed at 1000 rpm for 5 min. After centrifugation at 2000 rpm for 5 min, 900 μL of the MtBE supernatant was transferred to a glass culture tube. The supernatants were dried under N_2_ for 15 min at 40 °C. The residues were resuspended in 450 μL DDI H_2_O containing 20% acetonitrile, capped with a butyl rubber stopper, and mixed at 500 rpm for 15 min. Samples were immediately subjected to analysis via LC–MS/MS.

#### Accuracy, Precision, and Stability

Accuracy, precision, and stability measurements were made from samples prepared in the same fashion as the quality control samples (vide supra). Replicate injections (n = 5) were performed on a given sample on the same day (intraday), and independent samples were compared across multiple days (n = 3, interday). For freeze–thaw and long-term stability studies, 600 μL aliquots of unprocessed quality control samples were either subjected to three freeze–thaw cycles at −80 °C for at least 24 h, or they were frozen for 2 weeks at −80 °C before being thawed to room temperature for 30 min, extracted, and analyzed. Accuracy is reported as relative error, and precision is reported as relative standard deviation.

#### Recovery

Recovery was assessed by comparing (1) the measured concentration of each analyte when a mixture of the 22 molecules was spiked in before liquid–liquid extraction (termed pre-extraction) to (2) the measured concentration of each analyte when the same mixture of 22 molecules was spiked in after liquid–liquid extraction (termed postextraction). Samples were prepared in triplicate at 10 nM, 40 nM, 400 nM, or 800 nM, and processed according to the protocol for QC sample preparation (vide supra). Samples were subjected to analysis via LC–MS/MS immediately after preparation. Recovery was calculated as the percentage of the measured concentration of the pre-extraction spiked samples to the concentration of the postextraction spiked samples, according to eq 2. The results are reported as the mean ± S.E.M.

$$Recovery\left(\%\right)=\left(\frac{{Concentration}_{pre}}{{Concentration}_{post}}\right)\times 100\%$$ 2

### Donor Analysis

Fecal samples from individual donors (n = 10) were prepared in triplicate by adding 25 mg of lyophilized fecal material to 2.0 mL glass HPLC vials and processed according to the protocol for QC sample preparation (vide supra). Additionally, 50-fold and 10-fold dilutions were prepared by diluting either 2 μL of each sample with 98 μL, or 10 μL of each sample with 90 μL, respectively, of 5% aqueous acetonitrile containing IS. Samples were subjected to LC–MS/MS analysis immediately after preparation.

### LC–MS/MS Instrumentation and Parameters

Sample aliquots of 100 μL were added to a PlateOne round-bottom 96-well plate, sealed with NAL-96 Sealing Film (USA Scientific, Ocala, FL), and loaded into a Waters Sample Organizer refrigerated to 10 °C. A 4 μL aliquot of each sample was injected via a flow-through needle and Acquity I-Class Premier UPLC. The analytes were separated with a Waters Acquity Premier BEH C_18_ column (50 mm by 2.1 mm, 1.7 μm particle size), equipped with a VanGuard FIT BEH C_18_ guard column, and maintained at 50 °C. Solvent A was DDI H_2_O with 0.1% acetic acid, and Solvent B was acetonitrile with 0.1% acetic acid running at a flow rate of 0.500 mL/min. To provide an effective balance between signal intensity, peak shape, peak separation, and total run-time, the solvent gradient was 5% B to 40% B from 0–3.5 min, then ramped to 95% B over 0.1 min and held isocratic at 95% B until 4.15 min, and finally reduced to 5% B from 4.15–5.0 min.

Targeted mass spectrometry was performed with a Xevo TQ Absolute triple quadrupole mass spectrometer (Waters, Milford, MA, USA), operated in multiple-reaction monitoring (MRM) and negative electrospray ionization (ESI^—^) modes. Spectra were collected using MassLynx 4.2. The sample eluted through the capillary of the ZSpray source set to −0.80 kV and was sprayed toward the source cone set to 150 °C, with N_2_ desolvation gas flowing at 800 L/h and 600 °C. The precursor ion, fragment ion, cone voltage, and collision energy (Table 1 presents the quantifier ions and Table S2 presents the qualifier ions) were determined by injecting each analyte individually at 1.0 μM in acetonitrile, with each parameter selected to maximize the signal intensity for each analyte. Data were processed with the TargetLynx module.

**Table 1:** Mass Spectrometric Parameters^a^

	Q1 (*m*/*z*)	Q3 (*m*/*z*)	CV (V)	CE (eV)
**Phenolic Acids**				
3-Hydroxybenzoic Acid	136.90	92.96	20	10
3-(3-Hydroxyphenyl) propanoic Acid	164.99	105.98	20	10
3-(4-Hydroxyphenyl) propanoic Acid	165.00	58.97	30	10
4-Hydroxybenzoic Acid	136.90	92.94	20	10
Caffeic Acid	178.99	133.98	20	30
^13^C_3_–Caffeic Acid	181.97	136.99	10	20
Dihydrocaffeic Acid	180.98	136.99	10	10
Dihydroferulic Acid	195.05	120.99	20	30
Eudesmic Acid	211.02	151.93	20	20
Ferulic Acid	193.02	133.99	10	20
*para*-Coumaric Acid	163.00	92.99	10	30
Protocatechuic Acid	152.90	108.90	20	20
Sinapinic Acid	223.03	92.92	20	30
Syringic Acid	197.11	122.93	10	20
Vanillic Acid	166.96	107.95	10	20
Veratric Acid	180.99	136.98	30	10
**Lignans**				
Hydroxymatairesinol	373.34	173.01	50	30
Lariciresinol	359.18	328.98	10	10
Matairesinol	357.23	83.01	40	20
Pinoresinol	357.30	151.02	10	20
Secoisolariciresinol	361.19	164.97	40	30
*d*_6_-Secoisolariciresinol	367.16	167.92	20	30
**Enterolignans**				
Enterodiol	301.16	253.02	40	20
Enterolactone	297.16	106.99	20	30
^13^C_3_-Enterolactone	300.07	254.96	20	20

a Mass spectrometric parameters for the selective quantitation of all analytes and internal standards using multi-reaction monitoring in negative-ion mode. Q1 and Q3 are the precursor and fragment ions’ m/z, respectively. CV is the cone voltage, and CE is the collision energy.

## Results and Discussion

### Method Validation

#### Sensitivity

The chromatographic conditions were optimized to yield the lowest possible LLOQ for each analyte. The phenolic acids were quantified with LLOQs between 0.98 nM and 3.91 nM, and the lignans and enterolignans were quantified with LLOQs between 0.24 nM and 0.98 nM (Table 2). These results meet or exceed similar LC–MS/MS methods, where many phenolic acids have been reported with LLOQs between 19.5 nM^32^ and 800.4 nM,^33^ and many lignans have been reported with LLOQs between 0.068 nM^34^ and 3.91 nM.^28^ Carryover was negligible for all analytes.

**Table 2:** Method Sensitivity^a^

	LLOQ (nM)	ULOQ (nM)	R^2^
**Phenolic Acids**			
3-Hydroxybenzoic Acid	1.95	1000	0.998
3-(3-Hydroxyphenyl) propanoic Acid	1.95	1000	0.998
3-(4-Hydroxyphenyl) propanoic Acid	3.91	1000	0.997
4-Hydroxybenzoic Acid	3.91	1000	0.997
Caffeic Acid	0.98	1000	0.999
Dihydrocaffeic Acid	3.91	1000	0.998
Dihydroferulic Acid	1.95	1000	0.997
Eudesmic Acid	1.95	1000	0.998
Ferulic Acid	0.98	1000	0.996
*para*-Coumaric Acid	1.95	1000	0.998
Protocatechuic Acid	1.95	1000	0.998
Sinapinic Acid	0.98	1000	0.998
Syringic Acid	0.98	1000	0.999
Vanillic Acid	1.95	1000	0.998
Veratric Acid	1.95	1000	0.998
**Lignans**			
Hydroxymatairesinol	0.49	1000	0.997
Lariciresinol	0.98	1000	0.996
Matairesinol	0.25	1000	0.998
Pinoresinol	0.98	1000	0.999
Secoisolariciresinol	0.49	1000	0.997
**Enterolignans**			
Enterodiol	0.25	1000	0.998
Enterolactone	0.25	1000	0.997

a Summary of key sensitivity metrics for each compound. LLOQ is the lower limit of quantitation, ULOQ is the upper limit of quantitation, and R^2^ is the coefficient of determination from linear regression.

Various chromatographic solvent conditions were tested to achieve the best balance between peak shape and signal intensity. Neat solvents generally produced the largest signal intensities for all analytes, but they led to split-peaks for most phenolic acids (Figure 2). Adding 0.1% formic acid to the solvents caused a loss of signal intensity, such that the LLOQ for each analyte was 5–100 times higher than with either neat solvents or solvents containing 0.1% acetic acid. This was exemplified by the signal intensity for dihydroferulic acid, which was highest in neat solvents across the entire calibration range (Figure 2A), but the linearity was poor due to peak splitting (Figure 2B). Similarly, the signal for pinoresinol was obliterated when 0.1% formic acid was used in the chromatographic solvents, but the use of 0.1% acetic acid allowed for a strong, unambiguous peak at 0.31 nM (Figure 2C,D). These results support the finding of Song et al.: 0.1% acetic acid added to chromatographic solvents, instead of additives like formic acid, ammonium acetate, or ammonium fluoride, yields the best ion efficiencies and fragment ion coverage of various phenolic acids and lignin oligomers.^35^

**Figure 2:**
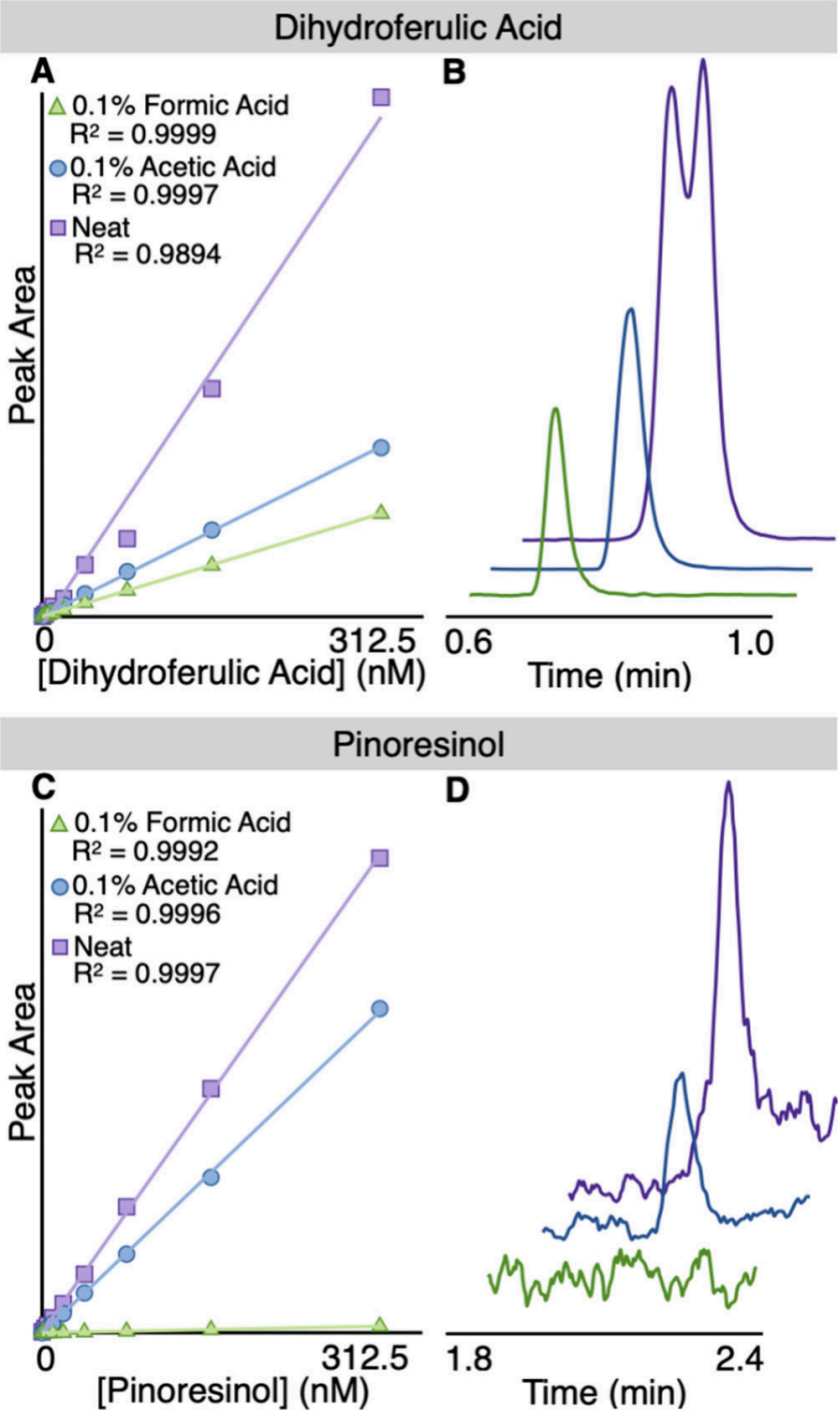
Chromatographic solvent additives impact peak shape and signal intensity. The mobile phase mixture of water and acetonitrile was tested alone (neat) or with additives (0.1% formic acid or 0.1% acetic acid). The representative effects of these additives on peak detection are depicted for (A,B) dihydroferulic acid and (C,D) pinoresinol. (A,C) Calibration curves and (B,D) extracted-ion chromatograms (dihydroferulic acid, 312.5 nM; pinoresinol, 0.31 nM) are shown.

#### Selectivity

The chromatographic conditions were optimized to selectively identify and quantify each analyte. While there was overlap in the elution of some phenolic acids, their distinct precursor and fragment ions allowed for unique identification. There was no overlap in the peaks for the lignans and enterolignans. The analytes 3-(3-hydroxyphenyl)­propanoic acid and pinoresinol display cross-signal contributions from other isobaric analytes, but baseline separation allows for accurate identification and quantification. The peak at 0.64 min that appears in the extracted-ion chromatogram of 3-(3-hydroxyphenyl)­propanoic acid results from a fragment of 3-(4-hydroxyphenyl)­propanoic acid because they have the same precursor *m*/*z*. To confirm this, each compound was injected separately. When 3-(4-hydroxyphenyl)­propanoic was injected alone, a signal appeared in the channel that is configured to detect 3-(3-hydroxyphenyl)­propanoic acid (Figure S1A). Similarly, pinoresinol showed a second peak at 2.3 min in its extracted-ion chromatogram that is the result of a cross-signal contribution from matairesinol (Figure S1B). Our method resolves this potentially confounding result, allowing for certainty in quantitation.

#### Matrix Effects

The matrix effects in this method are generally negligible, with most analytes displaying no more than 5% ion suppression or enhancement (Table S3). These results correspond to the matrix factor calculated by No̷rskov et al. in their detection of lignans from porcine fecal samples^36^ and by Sánchez-Patán et al. in their quantification of phenolic acids from fecal waters.^32^ Ion enhancements of 30.1% and 18.1% were observed for vanillic acid and matairesinol, respectively, at the 10 nM concentration, but the matrix effects on these analytes at the other QC concentrations were negligible. An ion enhancement effect of 13.1% was observed for enterodiol at the 10 nM concentration, whereas González-Domínguez observed between 38.2% and 51.1% ion suppression when enterodiol was spiked into a plasma matrix that was extracted with acetonitrile containing 1.5 M formic acid and 10 mM ammonium formate.^28^ The matrix effects that result from the method presented herein are negligible, allowing for the use of DDI H_2_O as a surrogate matrix because analyte-depleted fecal material is not available.^27^

Background contaminants imparted by polypropylene microcentrifuge tubes (MCTs) are a concern in LC–MS/MS analyses because they can lead to inaccurate quantitation, whether by ion suppression, ion enhancement, or cross-signal contribution. Multiple studies have found that MCTs can impart small molecule contaminants that are used as antistatic agents,^37^ plastic stabilizers,^38^ and plastic clarifying agents into biological samples.^39^ Canez and Li found that using MCTs for lipidomics imparted more contaminants than did using borosilicate glass.^40^ We have developed this method around the use of glass during sample preparation because the use of MCTs during liquid–liquid extraction gave rise to unpredictable, strong signals with the same precursor *m*/*z*, fragment *m*/*z*, and retention time as some of the analytes in the panel (Figure 3).

**Figure 3:**
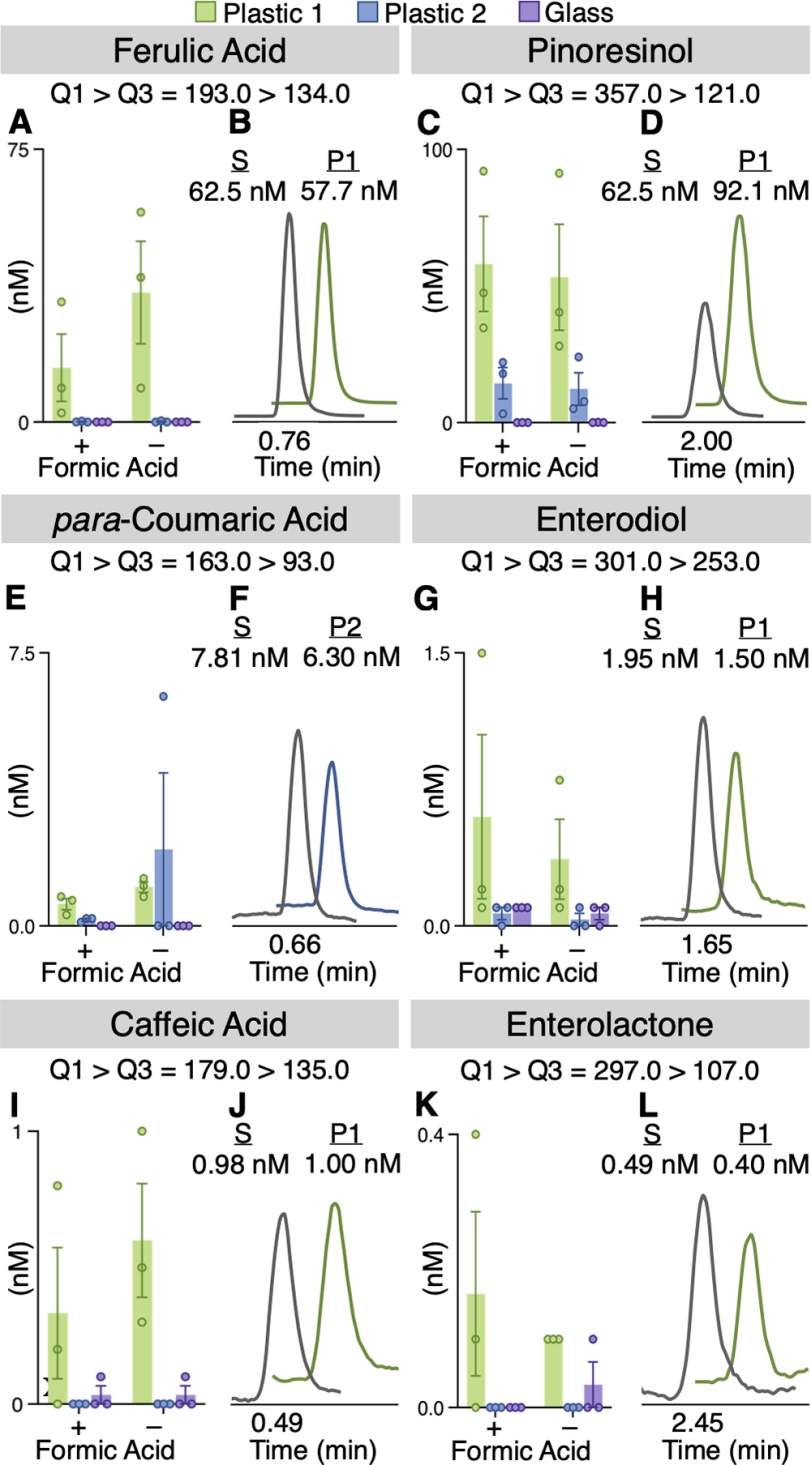
Extraction vessel impacts background signal. Leachate from two different brands of polypropylene microcentrifuge tubes (MCTs) upon liquid–liquid extraction of water, with or without 0.1% formic acid, using methyl *tert*-butyl ether. For (A,B) ferulic acid, (C,D) pinoresinol, (E,F) *para*-coumaric acid, (G,H) enterodiol, (I,J) caffeic acid, and (K,L) enterolactone, comparison of the observed concentrations of (A) ferulic acid, (C) pinoresinol, (E) *para*-coumaric acid, (G) enterodiol, (I) caffeic acid, or (K) enterolactone after extraction from either plastic or glass. Extracted ion chromatograms of microcentrifuge tube extracts are shown in comparison to standards of (B) ferulic acid, (D) pinoresinol, (F) *para*-coumaric acid, (H) enterodiol, (J) caffeic acid, or (L) enterolactone. *n* = 3 biological replicates; bars denote mean ± S.E.M. “S” represents the signal from a neat standard, and “P” represents the signal from the polypropylene MCT.

We tested an array of sample processing conditions with low binding MCTs from two different manufacturers against glass vials, each in triplicate. Leachate from Brand 1 MCTs produced signals in the ferulic acid channel between 2.5 nM and 57.7 nM, across all processing conditions (Figure 3A), which are between 2.6 and 58.9 times greater than the LLOQ for ferulic acid. The chromatograms in Figure 3B compare the absolute ferulic acid signal from a 62.5 nM neat standard to the signal observed when DDI H_2_O in MCTs from Brand 1 was extracted with MtBE. Leachate from MCTs also impacted pinoresinol quantification. Brand 1 MCTs produced signals in the pinoresinol channel that ranged from 28.0 nM to 92.1 nM, and Brand 2 MCTs produced signals that ranged from 3.3 nM to 24.1 nM (Figure 3C), which are between 3.4 and 94 times greater than the LLOQ for pinoresinol. Liquid–liquid extraction of DDI H_2_O with MtBE in MCTs caused interfering signals for other phenolic acids and enterolignans that ranged from 1.0 to 6.2 times their respective LLOQ (Figure 3E–L). The use of glass vials and tubes during sample preparation alleviated vessel interference in quantification.

#### Accuracy, Precision, and Stability

Accuracy, precision, and stability are crucial validation parameters because they help to establish that a method is robust and replicable. All analytes were quantified with accuracy and precision results within the acceptable tolerance (± 20% at the lowest QC concentration and ± 15% for all other concentrations), per FDA guidelines^27^ (Table 3). The results for most of the 22 analytes were within ± 10% relative error and relative standard deviation, which matches the results of similar methods.^32,36^ Sánchez-Patán et al. reported a −20.00% relative error for caffeic acid accuracy using stable-isotope labeled 4-hydroxy­benzoic acid as an internal standard.^32^ Conversely, the method reported herein substantially improved accuracy for caffeic acid (relative error range: −5.2%–0.8%) by using stable-isotope labeled caffeic acid as an internal standard. Additionally, Guadamuro et al. reported an accuracy of 117.41% for enterodiol,^41^ whereas the current method quantifies enterodiol with an accuracy between 95% and 105%.

**Table 3:** Robust Accuracy, Precision, and Recovery Metrics^a^

	Accuracy (RE%)				Precision (RSD%)				Recovery (%) Mean ± SEM			
	A	B	C	D	A	B	C	D	A	B	C	D
**Phenolic Acids**												
3-Hydroxybenzoic Acid	2.3	7.2	4.5	2.9	8.4	2.6	3.0	3.5	95.1 ± 2.1	103.0 ± 2.7	103.3 ± 2.5	104.8 ± 3.1
3-(3-Hydroxyphenyl) propanoic Acid	3.9	2.0	2.9	4.7	9.7	7.0	4.5	2.5	97.0 ± 5.0	90.0 ± 10.2	103.1 ± 2.4	104.9 ± 2.7
3-(4-Hydroxyphenyl) propanoic Acid	6.2	6.1	1.5	2.4	9.8	4.7	4.2	2.9	93.5 ± 4.9	98.7 ± 3.4	103.2 ± 3.0	101.5 ± 3.2
4-Hydroxybenzoic Acid	3.4	4.3	3.8	3.7	5.9	6.4	3.6	2.9	96.3 ± 4.6	97.3 ± 1.9	100.7 ± 2.6	104.7 ± 2.1
Caffeic Acid	–2.7	–5.2	–2.6	0.8	8.4	5.9	3.9	2.3	96.0 ± 2.7	96.4 ± 3.3	102.3 ± 2.0	102.2 ± 2.2
Dihydrocaffeic Acid	–1.5	–2.4	–4.2	–1.2	12.7	6.2	5.6	2.6	86.8 ± 4.1	99.2 ± 4.6	102.6 ± 1.9	104.5 ± 1.8
Dihydroferulic Acid	2.3	2.0	–2.9	–1.4	10.7	6.2	4.1	2.4	97.1 ± 5.8	102.1 ± 2.6	105.4 ± 2.4	107.1 ± 2.7
Eudesmic Acid	1.6	0.7	0.6	1.8	9.6	7.7	4.9	1.8	100.2 ± 5.6	98.8 ± 2.4	101.5 ± 1.7	102.2 ± 2.7
Ferulic Acid	2.8	0.1	–0.8	1.0	8.6	7.1	4.7	3.2	99.9 ± 5.3	99.9 ± 2.5	104.8 ± 2.3	104.3 ± 3.5
*para*-Coumaric Acid	3.7	–0.4	1.9	3.1	8.2	8.0	3.3	3.1	97.2 ± 2.2	99.1 ± 2.2	101.3 ± 2.6	102.9 ± 2.2
Protocatechuic Acid	0.0	–0.3	–0.5	3.8	9.9	5.2	5.3	5.0	92.7 ± 5.3	95.6 ± 2.1	99.7 ± 2.4	99.1 ± 2.9
Sinapinic Acid	0.4	–2.5	–1.4	0.4	7.6	5.3	4.0	4.8	100.3 ± 4.3	111.6 ± 1.8	106.9 ± 2.3	104.9 ± 3.3
Syringic Acid	3.6	–1.2	–2.2	0.5	8.7	6.4	4.6	6.0	98.9 ± 4.9	101.3 ± 3.5	106.0 ± 2.7	104.4 ± 2.4
Vanillic Acid	6.5	1.9	–1.6	0.1	8.0	6.5	5.1	5.8	105.3 ± 4.6	98.2 ± 3.4	104.4 ± 1.8	105.4 ± 2.0
Veratric Acid	3.6	1.1	–2.6	–0.5	11.6	8.5	4.2	2.6	106.6 ± 3.7	101.3 ± 2.6	103.3 ± 2.5	101.5 ± 2.7
**Lignans**												
Hydroxymatairesinol	2.0	2.1	–4.5	–6.9	11.5	4.0	5.2	4.2	103.7 ± 4.4	108.0 ± 3.2	104.1 ± 3.5	105.8 ± 1.9
Lariciresinol	3.3	3.6	–0.1	–5.1	9.2	5.9	8.8	6.8	99.5 ± 5.0	107.1 ± 2.9	107.7 ± 4.1	103.8 ± 1.8
Matairesinol	3.7	–2.9	1.3	2.8	4.7	4.5	4.2	3.9	95.1 ± 2.9	96.6 ± 1.7	95.8 ± 0.5	98.4 ± 1.4
Pinoresinol	0.1	–1.9	0.5	–0.1	6.6	4.7	3.0	3.6	102.4 ± 3.9	98.3 ± 2.5	93.0 ± 1.7	97.6 ± 1.5
Secoisolariciresinol	4.7	4.3	–1.0	–4.5	5.0	5.1	4.6	3.0	96.5 ± 2.3	100.0 ± 1.5	97.5 ± 2.3	100.2 ± 1.8
**Enterolignans**												
Enterodiol	–2.1	–4.8	–3.0	–0.7	5.2	4.5	3.5	4.7	93.8 ± 2.0	96.3 ± 1.9	96.9 ± 1.7	96.6 ± 1.4
Enterolactone	–2.1	–5.7	–3.7	–1.9	6.4	4.2	2.3	4.1	95.1 ± 1.9	101.5 ± 2.5	98.7 ± 1.1	100.9 ± 1.3

a Extracted analyte standards at A = 10 nM, B = 40 nM, C = 400 nM, or D = 800 nM. For accuracy and precision measurements, n = 3 independent sample sets, with n = 5 replicate injections per sample. Accuracy is reported as relative error (RE) and precision is reported as relative standard deviation (RSD). For recovery measurements, n = 3 independent sample sets, with n = 3 replicate injections per sample, and S.E.M. is standard error of the mean.

The accuracy and precision that this method affords was generally recapitulated in stability experiments **(** Table S4), with the sole exception that vanillic acid at the highest QC concentration (800 nM) produced relative errors of −18.4% and −19.3% (acceptable limit of ± 15%, per FDA guidelines),^27^ respectively, after three freeze–thaw cycles and after long-term storage at −80 °C.

#### Recovery

Recovery is an important validation parameter because it reflects the efficacy of the sample processing steps: if the recovery is too low, then improvements are required to ensure that the entire amount of each analyte is being measured, and if the recovery is too high, then there may be an inflationary impact from matrix-associated ion-enhancement phenomena.^42^ The recoveries for this method generally range from 95% to 105% (Table 3). The only analytes with recoveries less than 90% or greater than 110% were dihydrocaffeic acid (86.8%) and sinapinic acid (111.6%), both of which are recoveries that compare favorably with similar methods. The method reported by González-Domínguez et al. included recoveries for 3-(4-hydroxyphenyl)­propanoic acid, dihydroferulic acid, sinapinic acid, and vanillic acid of 120.0%, 114.2%, 119.5%, and 114.9%, respectively.^28^ Moreover, these authors reported recoveries of 71.7%, 78.5%, and 84.1% for enterodiol, matairesinol, and secoisolariciresinol, respectively.^28^ The method presented by No̷rskov et al. reported recoveries for enterodiol and enterolactone as not quantifiable and the recovery for pinoresinol as not detectable in the low QC samples.^36^ Lignan metabolites are generally present at low endogenous fecal concentrations, so recoveries that cluster around 100% via the current method demonstrate a significant improvement in their accurate detection and quantitation.

### Human Fecal Sample Analysis

Diets that are high in polyphenols, including phenolic acids and lignans, are associated with reduced risks for cancer^43^ and cardiovascular disease,^44^ which motivated the development of this assay. Thus, we tested the applicability of our method to the analysis of human fecal samples from 10 healthy donors (Table 4). The phenolic acid that reached the highest concentration across the fecal samples tested was the monophenol 3-(3-hydroxyphenyl)­propanoic acid (0.77–41.64 nmol/g of lyophilized fecal material). The measured abundance of 3-(3-hydroxyphenyl)­propanoic acid is corroborated by Gutiérrez-Díaz et al., who reported that, out of 30 phenolic acids, 3-(3-hydroxyphenyl)­propanoic acid was present in the fourth highest concentration in fecal samples from a cohort of Spanish adults who ate a Mediterranean diet.^45^ Gonthier et al. found that 3-(3-hydroxyphenyl)­propanoic acid was increased 15-fold in human urine following consumption of a polyphenol-rich diet,^46^ and Lu et al. found that 3-(3-hydroxyphenyl)­propanoic acid was a metabolic end product when human fecal inoculum was fermented with ferulic acid.^47^ Collectively, our finding that 3-(3-hydroxyphenyl)­propanoic acid is an abundant phenolic acid in human fecal samples aligns with existing measurements of this analyte, supporting the utility of our method.

**Table 4:** Analytes Are Effectively Measured in Human Fecal Samples^a^

	Human Fecal Sample Donor									
	1	2	3	4	5	6	7	8	9	10
**Phenolic acids**										
3-Hydroxybenzoic Acid	1.33 ± 0.03	6.89 ± 0.37	4.10 ± 0.05	2.03 ± 0.12	1.26 ± 0.02	26.92 ± 3.07	3.27 ± 0.42	3.39 ± 0.11	2.64 ± 0.05	8.06 ± 0.85
3-(3-Hydroxyphenyl) propanoic Acid	2.85 ± 0.07	17.92 ± 1.49	4.34 ± 0.29	27.69 ± 1.11	29.83 ± 4.89	41.64 ± 2.22	4.22 ± 1.55	9.59 ± 1.60	0.77 ± 0.22	9.10 ± 0.83
3-(4-Hydroxyphenyl) propanoic Acid	0.61 ± 0.10	9.05 ± 0.81	2.18 ± 0.09	6.23 ± 0.38	1.38 ± 0.17	3.31 ± 0.16	4.20 ± 0.72	7.21 ± 2.36	5.80 ± 0.56	1.47 ± 0.04
4-Hydroxybenzoic Acid	1.93 ± 0.03	3.14 ± 0.12	5.58 ± 0.17	4.57 ± 0.31	0.82 ± 0.04	17.90 ± 3.15	2.24 ± 0.30	3.69 ± 0.19	2.49 ± 0.11	5.19 ± 0.21
Caffeic Acid	0.65 ± 0.04	3.66 ± 0.91	0.94 ± 0.02	0.47 ± 0.01	0.50 ± 0.19	3.31 ± 0.76	0.50 ± 0.17	0.64 ± 0.06	0.52 ± 0.07	0.54 ± 0.01
Dihydrocaffeic Acid	0.31 ± 0.01	5.65 ± 0.79	1.04 ± 0.08	0.61 ± 0.10	0.49 ± 0.05	3.23 ± 0.09	0.50 ± 0.05	0.76 ± 0.02	0.63 ± 0.07	2.04 ± 0.04
Dihydroferulic Acid	2.83 ± 0.21	6.56 ± 0.68	0.66 ± 0.02	0.46 ± 0.05	1.73 ± 0.03	1.03 ± 0.04	2.36 ± 0.44	0.54 ± 0.05	0.40 ± 0.06	0.37 ± 0.01
Eudesmic Acid	0.02 ± 0.01	0.15 ± 0.01	0.04 ± 0.01	0.05 ± 0.01	2.48 ± 0.05	nq	0.14 ± 0.02	0.05 ± 0.03	0.22 ± 0.12	nq
Ferulic Acid	5.46 ± 1.06	11.43 ± 2.04	0.80 ± 0.05	0.89 ± 0.12	2.22 ± 0.24	3.02 ± 0.26	1.82 ± 0.32	1.05 ± 0.17	0.87 ± 0.09	0.44 ± 0.01
*para*-Coumaric Acid	0.81 ± 0.02	3.71 ± 1.21	0.59 ± 0.05	0.71 ± 0.11	0.18 ± 0.02	3.68 ± 0.36	1.27 ± 0.15	3.61 ± 1.65	4.28 ± 0.84	0.27 ± 0.02
Protocatechuic Acid	0.64 ± 0.03	8.37 ± 0.08	0.72 ± 0.03	1.80 ± 0.09	0.19 ± 0.10	2.59 ± 0.30	1.45 ± 0.11	1.50 ± 0.12	3.31 ± 0.19	1.21 ± 0.02
Sinapinic Acid	0.09 ± 0.01	0.45 ± 0.04	0.09 ± 0.00	0.07 ± 0.00	0.05 ± 0.00	0.62 ± 0.06	0.18 ± 0.03	0.19 ± 0.05	0.11 ± 0.00	0.05 ± 0.00
Syringic Acid	0.07 ± 0.00	0.60 ± 0.03	0.27 ± 0.01	0.17 ± 0.01	nq	0.18 ± 0.00	1.06 ± 0.13	0.38 ± 0.04	1.08 ± 0.04	0.30 ± 0.02
Vanillic Acid	nq	1.00 ± 0.03	0.25 ± 0.01	1.01 ± 0.05	0.15 ± 0.04	1.66 ± 0.22	0.20 ± 0.03	0.24 ± 0.02	0.17 ± 0.01	0.12 ± 0.02
Veratric Acid	0.03 ± 0.00	0.06 ± 0.00	0.03 ± 0.00	0.03 ± 0.00	0.10 ± 0.01	0.06 ± 0.00	0.02 ± 0.00	0.04 ± 0.01	0.11 ± 0.04	nq
**Lignans**										
Hydroxymatairesinol	nd	nq	nd	0.24 ± 0.03	0.01 ± 0.00	0.01 ± 0.00	nd	0.01 ± 0.01	0.01 ± 0.00	0.03 ± 0.00
Lariciresinol	0.01 ± 0.00	0.02 ± 0.00	0.01 ± 0.00	0.03 ± 0.00	0.04 ± 0.00	0.04 ± 0.00	0.02 ± 0.00	0.08 ± 0.01	0.02 ± 0.00	0.10 ± 0.00
Matairesinol	0.03 ± 0.00	0.03 ± 0.00	0.01 ± 0.00	0.17 ± 0.01	0.18 ± 0.01	0.13 ± 0.02	0.02 ± 0.01	0.01 ± 0.00	0.02 ± 0.00	0.02 ± 0.00
Pinoresinol	nq	0.03 ± 0.01	0.03 ± 0.01	0.03 ± 0.02	0.15 ± 0.09	0.06 ± 0.01	0.03 ± 0.01	0.09 ± 0.02	0.37 ± 0.04	0.03 ± 0.01
Secoisolariciresinol	nd	0.01 ± 0.00	0.01 ± 0.00	0.01 ± 0.00	0.02 ± 0.00	0.01 ± 0.00	0.01 ± 0.00	nq	0.01 ± 0.00	0.01 ± 0.00
**Enterolignans**										
Enterodiol	1.43 ± 0.02	1.03 ± 0.13	0.68 ± 0.07	6.71 ± 0.69	0.52 ± 0.01	1.75 ± 0.09	0.94 ± 0.11	0.72 ± 0.04	1.04 ± 0.08	0.55 ± 0.01
Enterolactone	4.24 ± 0.05	6.30 ± 0.61	2.47 ± 0.17	87.78 ± 11.14	1.24 ± 0.04	40.94 ± 1.61	12.21 ± 4.61	2.38 ± 0.59	0.65 ± 0.17	5.58 ± 0.09

a Measured concentrations of phenolic acids, lignans, and enterolignans in human fecal samples (n = 10 independent donors). Values are mean ± S.E.M. (nmol/g), normalized to the dry mass of each sample; n = 3 technical replicates per donor. ″nd″ denotes not detected, and ″nq″ denotes a measurement below the lower limit of quantitation, therefore not quantifiable.

In addition to accurately quantifying analytes in high fecal concentrations, one strength of this method is that its improved sensitivity allows for accurately quantifying molecules in low fecal concentrations. Quantification of lignans found them to be in relatively low abundance (0.01–0.37 nmol/g of lyophilized fecal material). While many existing methods either do not report concentrations for some lignans or they report that some lignans were not detected,^36,48^ the method reported here enabled quantification of 5 lignans and 2 enterolignans in fecal samples from 10 donors. The enterolignans were up to 237-fold more abundant (enterodiol, 0.52–6.71 nmol/g of lyophilized fecal material; enterolactone, 0.65–87.78 nmol/g of lyophilized fecal material) than the lignans (Table 4). The increased abundance of enterolignans in comparison with lignans aligns with known biosynthetic pathways. Specifically, the 5 lignans in our panel are biochemical precursors to the enterolignans via processing by human gut bacteria.^23^ Kasimir et al. cultured bacteria from pig ceca with uniformly ^13^C-labeled lignin and found that lignin-derived secoisolariciresinol began to be transformed to enterolactone within 2 h and was fully depleted within 24 h.^49^

The validated LC–MS/MS method described here has been designed to be simple and robust, with the goal of facilitating gut microbiome research that seeks to elucidate the roles of polyphenolic metabolites in human health and disease. This method advances the field of phenolic acid, lignan, and enterolignan quantitation in human fecal samples by simultaneously quantifying these diet- and gut microbiota-derived metabolites with a short analytical cycle of 5 min per injection. Importantly, this method allows for direct comparisons between individuals and across time because the analyte concentrations are normalized to the mass of lyophilized fecal material, thereby removing any confounding effects imparted by differences in water content. This method enables reliable quantitation of many lignans, which are typically in low abundance in human fecal samples, whereas many existing methods report them as either not detected or not quantifiable. Taken together, this accurate and sensitive method to simultaneously quantify a panel of health-promoting molecules will facilitate future studies that quantify and tailor their gut bacterial biosynthesis for disease prevention.

## Supplementary Material



## Notes

Common and IUPAC names of analytes included in this panel; extracted-ion chromatograms for 3-(3-hydroxyphenyl)­propanoic acid and pinoresinol show cross-signal contributions; mass spectrometric parameters for the qualifier ions of each analyte; summary of results for validation experiments to investigate matrix effects; summary of results for validation experiments to investigate stability (PDF)
