# Supplementary material for: A Validated LC–MS/MS Method for Quantifying Phenolic Acids, Lignans, and Enterolignans from Human Fecal Samples
Source: Acs Nutr Sci. 2026 Jun 11;1(4):356–66. doi: 10.1021/acsnutrsci.6c00063 (PMC13394526; doi:10.1021/acsnutrsci.6c00063)
Supplement: Supplementary file 1 [file ns6c00063_si_001.pdf]

Supporting Information: A Validated LC–MS/MS Method for Quantifying Phenolic Acids, Lignans, and Enterolignans from Human Fecal Samples

Christopher A. Dicksion[1], Darrian N. Chao[1], Jonathan D. Rickmeyer[2], and Elizabeth N. Bess[1,3]*

[1]Department of Chemistry, University of California, Irvine, California 92617, United States

[2]School of Pharmacy and Pharmaceutical Sciences, University of California, Irvine, California 92617, United States

[3]Department of Molecular Biology and Biochemistry, University of California, Irvine, California 92617, United States

*Corresponding author: Elizabeth N. Bess, Departments of Chemistry and Molecular Biology & Biochemistry, University of California, Irvine, 1102 Natural Sciences II, Irvine, CA 92617, USA.

E-mail: elizabeth.bess@uci.edu

Table S1: Common and IUPAC names for phenolic acids, lignans, and enterolignans included in the method, as proposed by Kay, et al.[1]

Common Name	IUPAC Name
Phenolic Acids	
Caffeic Acid	3-(3,4-Dihydroxyphenyl)-2-propenoic acid
Dihydrocaffeic Acid	3-(3,4-Dihydroxyphenyl)-2-propanoic acid
Dihydroferulic Acid	3-(4-hydroxy-3-methoxyphenyl)-2-propanoic acid
Eudesmic Acid	3,4,5-Trimethoxybenzoic acid
Ferulic Acid	3-(4-hydroxy-3-methoxyphenyl)-2-propenoic acid
para-Coumaric Acid	3-(4-Hydroxyphenyl)-2-propenoic acid
Protocatechuic Acid	3,4-Dihydroxybenzoic acid
Sinapinic Acid	3-(4-Hydroxy-3,5-dimethoxyphenyl)-2-propenoic acid
Syringic Acid	4-Hydroxy-3,5-dimethoxybenzoic acid
Vanillic Acid	4-Hydroxy-3-methoxybenzoic acid
Veratric Acid	3,4-Dimethoxybenzoic acid
Lignans	
Hydroxymatairesinol	(3*R*,4*R*)-4-[(*S*)-Hydroxy(4-hydroxy-3-methoxyphenyl)methyl]-3-[(4-hydroxy-3-methoxyphenyl)methyl]oxolan-2-one
Lariciresinol	4-[(2*S*,3*R*,4*R*)-4-[(4-Hydroxy-3-methoxyphenyl)methyl]-3-(hydroxymethyl)oxolan-2-yl]-2-methoxyphenol
Matairesinol	(3*R*,4*R*)-3,4-Bis[(4-hydroxy-3-methoxyphenyl)methyl]oxolan-2-one
Pinoresinol	4-[(3S,3aR,6S,6aR)-6-(4-hydroxy-3-methoxyphenyl)-1,3,3a,4,6,6a-hexahydrofuro[3,4-c]furan-3-yl]-2-methoxyphenol
Secoisolariciresinol	(8*R*,8′*R*)-3,3′-Dimethoxylignane-4,4′,9,9′-tetrol
Enterolignans	
Enterodiol	(2*R*,3*R*)-2,3-Bis[(3-hydroxyphenyl)methyl]butane-1,4-diol
Enterolactone	(3*R*,4*R*)-3,4-Bis[(4-hydroxyphenyl)methyl]oxolan-2-one

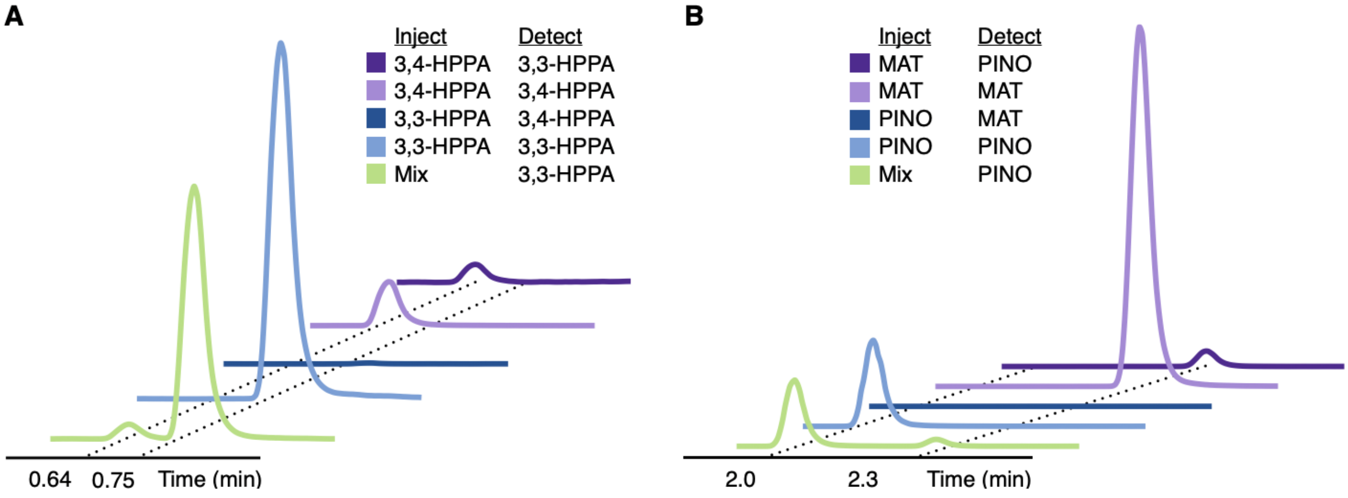

Figure S1: Cross-Signal Contributions Across Analytes. (A) Overlaid extracted-ion chromatograms of 3-(3-hydroxyphenyl)propanoic acid (3,3-HPPA) and 3-(4-hydroxyphenyl)propanoic acid (3,4-HPPA), showing that the peak at 0.64 minutes in the extracted-ion chromatogram for 3,3-HPPA from a mixed sample of 3,3-HPPA and 3,4-HPPA is a cross-signal contribution from 3,4-HPPA. **(B)** Overlaid extracted-ion chromatograms of pinoresinol (PINO) and matairesinol (MAT), showing that the peak at 2.3 minutes in the extracted-ion chromatogram for pinoresinol from a mixed sample of pinoresinol and matairesinol is a cross-signal contribution from matairesinol. "Detect" means that the mass spectrometer was set to the parameters for detection of the analyte listed in the "Detect" column.

Table S2: Mass Spectrometric Parameters for Qualifier Ion Transitions. Mass spectrometric parameters for the selective qualification of all analytes and internal standards using multi-reaction monitoring in negative-ion mode. Q1 and Q3 are the precursor and fragment ions' m/z, respectively. CV is the cone voltage, and CE is the collision energy.

**Phenolic Acids**	Q1 (m/z)	Q3 (m/z)	CV (V)	CE (eV)
3-Hydroxybenzoic Acid	136.90	99.85	20	20
3-(3-Hydroxyphenyl) propanoic Acid	164.99	121.00	20	10
3-(4-Hydroxyphenyl) propanoic Acid	165.00	93.00	20	10
4-Hydroxybenzoic Acid	136.90	99.85	20	20
Caffeic Acid	178.99	135.01	20	20
Dihydrocaffeic Acid	180.98	58.96	10	20
Dihydroferulic Acid	195.05	135.99	20	20
Eudesmic Acid	211.02	166.94	20	10
Ferulic Acid	193.02	177.95	10	10
para-Coumaric Acid	163.00	118.99	10	20
Protocatechuic Acid	152.90	107.95	40	20
Sinapinic Acid	223.03	120.96	20	30
Syringic Acid	197.11	94.92	10	30
Vanillic Acid	166.96	151.92	10	10
Veratric Acid	180.99	121.96	30	20
**Lignans**				
Hydroxymatairesinol	373.34	355.02	50	20
Lariciresinol	359.18	159.95	10	40
Matairesinol	357.23	122.00	40	40
Pinoresinol	357.30	135.97	10	40
Secoisolariciresinol	361.19	120.97	40	50
**Enterolignans**				
Enterodiol	301.16	105.97	40	30
Enterolactone	297.16	253.03	20	20

Table S3: Matrix Effects. Matrix effects impacting analytes in human fecal samples spiked with standards at A = 10 nM, B = 40 nM, C = 400 nM, or D = 800 nM compared to mixtures of analytes in neat solutions at the same nominal concentrations. Results are the percent similarity of the spiked analyte response to the neat analyte response. Values are mean ± S.E.M.; n = 3 independent sample sets, with n = 3 replicate injections.

**Phenolic Acids**	A (%)	B (%)	C (%)	D (%)
3-Hydroxybenzoic Acid	95.7 ± 4.6	98.2 ± 1.5	99.0 ± 0.9	96.9 ± 1.0
3-(3-Hydroxyphenyl)propanoic Acid	95.0 ± 5.4	95.5 ± 8.1	88.3 ± 0.7	91.9 ± 2.7
3-(4-Hydroxyphenyl)propanoic Acid	97.6 ± 3.7	100.9 ± 6.2	95.9 ± 1.4	94.6 ± 1.7
4-Hydroxybenzoic Acid	103.6 ± 4.2	104.5 ± 0.6	103.2 ± 1.2	100.8 ± 0.5
Caffeic Acid	90.4 ± 9.2	95.7 ± 1.7	100.2 ± 1.2	103.1 ± 1.2
Dihydrocaffeic Acid	96.6 ± 9.2	101.0 ± 1.7	100.6 ± 1.6	101.3 ± 1.0
Dihydroferulic Acid	99.5 ± 3.3	91.3 ± 2.6	95.1 ± 1.1	94.0 ± 1.1
Eudesmic Acid	102.2 ± 2.9	92.1 ± 1.6	100.9 ± 1.0	98.8 ± 1.1
Ferulic Acid	102.7 ± 2.8	91.3 ± 1.6	94.9 ± 1.3	94.7 ± 1.1
para-Coumaric Acid	104.4 ± 2.6	93.5 ± 1.2	92.0 ± 1.0	93.4 ± 1.2
Protocatechuic Acid	106.0 ± 3.2	101.8 ± 2.1	102.1 ± 0.8	101.0 ± 1.3
Sinapinic Acid	97.4 ± 3.6	99.2 ± 1.4	100.5 ± 1.4	98.0 ± 0.8
Syringic Acid	106.6 ± 4.8	98.0 ± 1.3	95.3 ± 1.0	98.4 ± 2.1
Vanillic Acid	130.1 ± 13.6	96.3 ± 3.3	94.7 ± 1.3	95.0 ± 0.9
Veratric Acid	92.7 ± 5.6	90.3 ± 1.7	93.1 ± 1.2	96.7 ± 1.1
**Lignans**				
Hydroxymatairesinol	89.2 ± 4.8	88.4 ± 1.5	86.9 ± 1.6	96.3 ± 1.8
Lariciresinol	95.2 ± 3.1	92.9 ± 2.3	87.8 ± 1.6	96.3 ± 2.1
Matairesinol	118.9 ± 2.9	100.6 ± 0.7	101.6 ± 1.0	100.9 ± 1.1
Pinoresinol	99.7 ± 4.6	101.0 ± 2.3	102.9 ± 0.7	99.3 ± 1.0
Secoisolariciresinol	98.5 ± 2.0	96.4 ± 2.8	93.2 ± 1.5	100.5 ± 1.0
**Enterolignans**				
Enterodiol	113.1 ± 3.3	105.7 ± 1.4	106.3 ± 1.2	103.0 ± 0.8
Enterolactone	103.2 ± 4.5	98.9 ± 2.8	100.6 ± 0.8	99.1 ± 0.6

Table S4: Freeze-Thaw and Long-Term Stability. Accuracy and precision of extracted analytes at A = 10 nM, B = 40 nM, C = 400 nM, or D = 800 nM, either subjected to three freeze-thaw cycles of at least 24 hours or stored at -80 °C for two weeks. Accuracy is reported as relative error (RE) and precision is reported as relative standard deviation (RSD); n = 3 independent sample sets, with n = 5 replicate injections per sample.

	Freeze-Thaw Stability								Long-Term Stability							
	Accuracy (RE%)				Precision (RSD%)				Accuracy (RE%)				Precision (RSD%)			
**Phenolic Acids**	A	B	C	D	A	B	C	D	A	B	C	D	A	B	C	D
3-Hydroxybenzoic Acid	13.7	10.7	-2.9	-5.8	5.6	3.2	2.8	2.6	15.2	7.0	-5.4	-7.5	3.0	6.6	3.4	3.8
3-(3-Hydroxyphenyl) propanoic Acid	10.4	8.1	-0.2	0.3	6.2	4.5	2.1	2.7	12.1	9.3	-5.0	-2.5	5.3	3.5	2.9	3.5
3-(4-Hydroxyphenyl) propanoic Acid	9.2	8.4	1.5	0.7	7.4	3.5	2.4	2.6	11.3	9.2	-3.8	-0.9	5.4	3.3	2.2	3.1
4-Hydroxybenzoic Acid	14.9	9.5	-0.4	-1.3	4.1	3.2	2.6	2.5	17.9	6.2	-3.8	-2.6	4.6	6.6	2.4	3.6
Caffeic Acid	-6.7	-5.0	-3.9	1.5	5.0	3.2	2.6	2.2	-5.2	-4.5	-4.4	2.4	4.1	2.3	2.1	2.4
Dihydrocaffeic Acid	-9.8	-11.0	-10.3	-5.7	11.9	8.0	3.1	2.5	-9.3	-13.3	-12.6	-7.4	12.0	4.6	2.7	3.3
Dihydroferulic Acid	14.3	7.9	-3.6	-2.3	3.4	4.0	3.7	5.1	13.1	7.6	-6.1	-4.1	4.8	4.6	3.6	3.7
Eudesmic Acid	14.2	10.6	1.2	0.2	3.6	3.6	6.3	5.8	14.2	9.7	-1.0	-0.6	3.2	3.1	5.9	5.6
Ferulic Acid	8.1	7.8	1.9	1.0	4.3	4.1	3.4	2.6	12.6	7.0	-2.4	0.1	2.1	3.9	4.1	3.9
para-Coumaric Acid	2.4	7.6	3.7	2.7	8.3	3.2	2.6	2.2	7.6	4.7	-3.6	0.7	6.0	3.7	3.3	2.5
Protocatechuic Acid	5.6	7.4	-0.1	2.7	4.9	3.7	3.3	3.1	10.0	6.0	-5.6	0.6	6.6	5.1	3.5	3.4
Sinapinic Acid	-9.9	-9.2	-0.2	0.9	8.1	4.5	5.9	5.5	-11.8	-9.0	-4.6	-2.0	8.3	5.1	5.5	6.5
Syringic Acid	7.6	1.9	-5.1	-4.9	5.7	5.5	3.0	4.1	9.3	3.0	-9.4	-5.8	6.7	6.5	4.1	4.5
Vanillic Acid	12.7	6.7	-15.5	-18.4	5.4	4.6	12.2	6.9	14.0	-4.2	-14.8	-19.3	3.1	18.7	4.6	3.5
Veratric Acid	11.1	10.9	-11.7	-10.9	4.8	3.7	3.7	2.1	10.6	3.5	-11.7	-11.6	3.9	8.9	2.5	2.9
**Lignans**																
Hydroxymatairesinol	-15.6	-10.3	-6.8	-1.7	5.8	4.1	3.2	4.2	-15.2	-10.8	-8.4	-3.8	5.6	4.8	4.2	3.7
Lariciresinol	-2.3	1.2	3.2	5.9	7.7	7.1	3.8	3.1	-1.0	4.0	2.6	4.5	9.3	5.5	5.3	3.8
Matairesinol	-17.5	-9.9	2.5	8.1	2.2	1.9	1.9	2.5	-14.7	-5.5	2.1	6.7	3.5	3.4	1.8	1.8
Pinoresinol	-5.6	0.3	6.1	8.0	4.8	5.2	3.6	3.1	-3.3	0.3	5.7	8.9	8.0	4.8	4.6	4.0
Secoisolariciresinol	-8.3	-2.9	-1.0	0.7	5.4	4.8	4.7	2.6	-6.2	-2.8	-1.4	-0.8	5.6	3.0	4.6	2.5
**Enterolignans**																
Enterodiol	-12.5	-5.8	3.0	5.1	5.2	2.9	3.4	2.8	-11.7	-4.5	3.1	4.4	4.4	3.8	4.2	2.1
Enterolactone	-9.0	-0.6	-0.1	0.7	2.0	3.0	2.6	2.4	-5.9	0.8	-0.4	1.2	1.8	3.7	3.8	2.5

References for Supporting Information

(1) Kay, C.D.; Clifford, M.N.; Mena, P.; McDougall, G.J.; Andrés-Lacueva, C.; Cassidy, A.; Del Rio, D.; Kuhnert, N.; Manach, C.; Pereira-Caro, G.; Rodriguez-Mateos, A.; Scalbert, A.; Tomás-Barberán, F.; Williamson, G.; Wishart, D.S.; Crozier, A. Recommendations for standardizing nomenclature for dietary (poly)phenol catabolites. *Am. J. Clin. Nutr*. **2020**, 112(4):1051-1068.
